# Procedure for Reliable and Long-Lasting Ex Vivo Recordings of Sciatic Nerve Activity in Mice

**DOI:** 10.21769/BioProtoc.5233

**Published:** 2025-03-05

**Authors:** Shani Berkowitz, Zehavit Goldberg, Amir Dori, Nicola Maggio, Efrat Shavit-Stein, Jérôme Joël Devaux

**Affiliations:** 1 Department of Neurology, The Chaim Sheba Medical Center, Ramat Gan, Israel; 2 Department of Neurology and Neurosurgery, Faculty of Medical & Health Sciences, Tel Aviv University, Tel Aviv, Israel; 3 Talpiot Medical Leadership Program, The Chaim Sheba Medical Center, Ramat Gan, Israel; 4 Sagol School of Neuroscience, Tel Aviv University, Tel Aviv, Israel; 5 The TELEM Rubin Excellence in Biomedical Research Program, The Chaim Sheba Medical Center, Ramat Gan, Israel; 6 Institut de Génomique Fonctionnelle, Université de Montpellier, CNRS, INSERM, Montpellier, France

**Keywords:** Sciatic nerve, Electrophysiology, Compound action potential, Neuronal excitability, Conduction, Neuropathy, Myelin, Node of Ranvier

## Abstract

Changes in neuronal conduction are common in disease states affecting peripheral nerves. These alterations can significantly impact nerve function and lead to sensorimotor disabilities. In vivo electromyography recording is a well-established electrophysiological method that has been used for decades to assess sensory and motor functions in the nervous system. Nerve studies are challenging to conduct in vivo in rodents, and the involvement of muscle activity makes it difficult to isolate and assess nerve function independently. This protocol provides a comprehensive guide for accurate ex vivo sciatic nerve dissection and handling from mice. It includes the creation of a three-compartment chamber and the establishment of electrophysiological protocols, which enable differential recordings and the analysis of compound action potentials from various nerve fibers. This setup allows researchers to study the specific effects of drugs and pathologies on nerves from a mechanistic perspective. The setup is a stand-alone apparatus that does not require the use of suction electrodes and the maintenance of negative pressure, which can affect the signal-to-noise ratio and recording stability.

Key features

• Sciatic nerve electrophysiology recordings from mice.

• Allows for testing of disease model effects.

• Ex vivo setup enables accurate pharmacological tests.

• User-friendly software acquisition and analysis of compound action potential response.

## Graphical overview


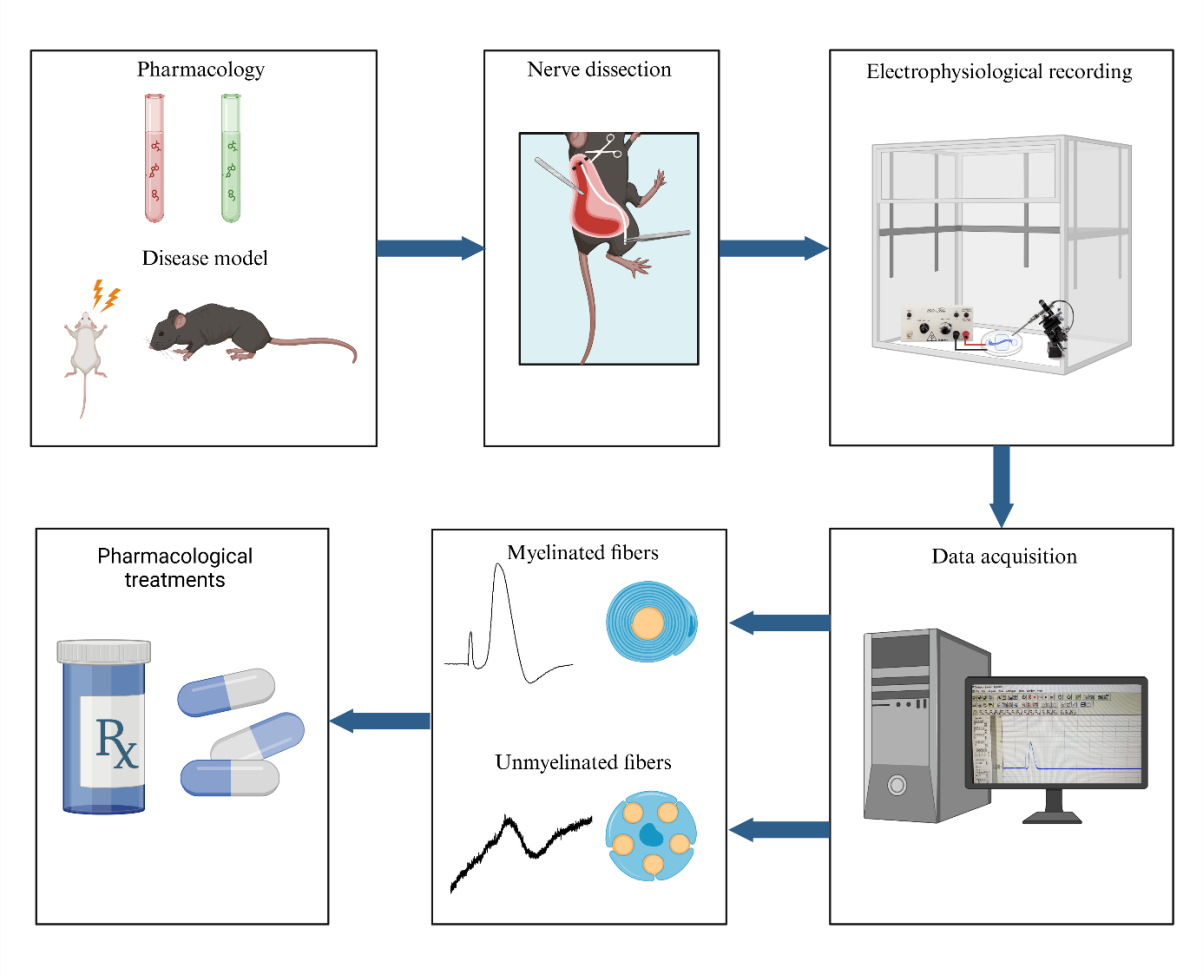


## Background

Peripheral nerve function is compromised in a range of disorders, including diabetic neuropathy and autoimmune conditions like Guillain-Barré syndrome and chronic inflammatory demyelinating polyneuropathy, as well as hereditary diseases such as Charcot-Marie-Tooth disease [1–4]. Injuries, infections (e.g., Lyme disease, *Mycobacterium leprae*), vitamin deficiency, and intoxications (e.g., heavy metals) may also lead to nerve damage [3,5,6]. Depending on which nerves and nerve fibers are affected, the symptoms can include pain, numbness, tingling, and weakness, and can alter sensory, motor, or autonomic functions in the body [7]. Nociceptors detect harmful stimuli like extreme temperature, pressure, or injury-related chemicals in the skin. The signal is then converted into electrical impulses sent to the brain, shaping the diverse qualities of pain [8,9].

In vivo electromyography is a long-established method for assessing sensory and motor functions in the peripheral nervous system [10,11]. Conducting nerve studies in live rodents poses several challenges. Muscle activity is often involved, making it difficult to isolate and assess nerve function independently—an important distinction when evaluating effects on nerves vs. muscles. Another challenge with in vivo electrophysiological setups is the impact of anesthetics and blood perfusion, which can interfere with direct pharmacological effects or influence nerve activity. Maintaining physiological temperature is also essential in these setups, as temperature strongly modulates nerve conduction. Finally, the use of a smaller device and low-volume chamber helps minimize noise within the system, improving control and accuracy.

**Figure 1.**
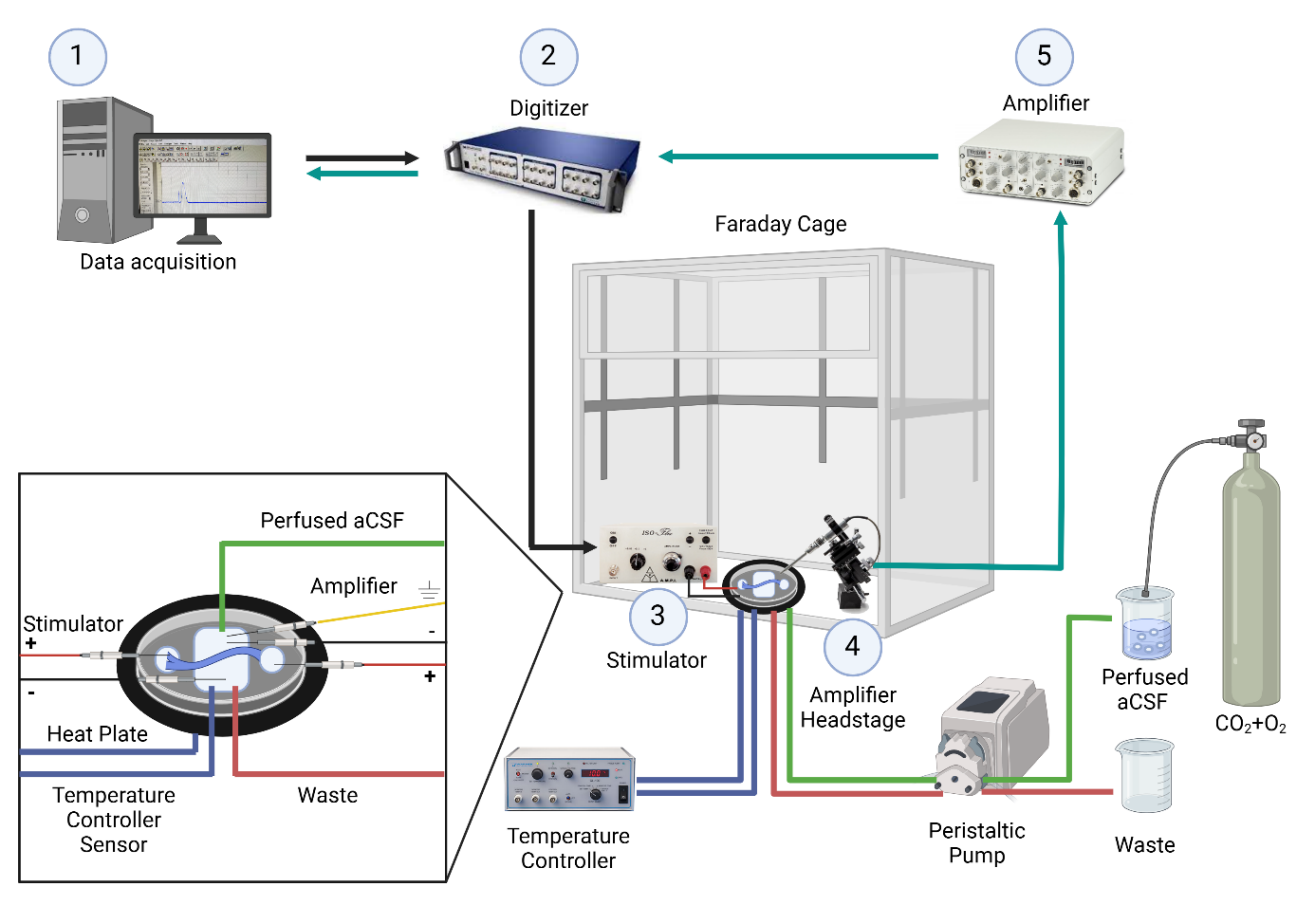
Electrophysiology setup for ex-vivo sciatic nerve recordings. The nerve is placed in a temperature-controlled chamber with continuously perfused artificial cerebrospinal fluid. 1. Data is acquired and analyzed using Clampex software. 2. A command signal is sent from the computer to the digitizer, which is then relayed from the digitizer to the stimulator. 3. Nerve stimulation occurs through a pair of electrodes connected to the stimulator and placed in the left well (positive electrode) and middle chamber (negative electrode). 4. The signal is acquired through the amplifier headstage connected to a pair of recording electrodes placed in the middle chamber (negative electrode) and the well on the right side (positive electrode). 5. The signal is then amplified. After amplification, the signal is sent to the digitizer and back to the computer. Created with BioRender.com.

An ex vivo electrophysiology setup for the isolated sciatic nerve is a vital tool in neuroscience research, offering significant insights into nerve function and pathology. The use of suction electrodes, which gently capture nerve fibers to record action potentials is increasingly used to study ex vivo peripheral nerve function [12,13]. However, suction electrodes can be technically difficult to prepare and use. Also, potential amplitude and recording stability strongly depend on both the quality of the suction maintenance and the nerve/electrode resistance [14]. An alternative is the use of a Vaseline-sealed three-chamber-compartment recording technique (Figure 1). This setup enables precise recording of electrical activity from the isolated mouse sciatic nerve, facilitating the study of various disease models and the effects of pharmacological manipulations. Numerous animal models are available to investigate myelin biology, conduction, and the pathological mechanisms underlying conduction defects in peripheral neuropathies. While electromyographic recording offers limited insights into both myelinated and unmyelinated fiber populations, ex vivo recordings of nerve trunks provide a valuable opportunity to examine specific alterations in axonal populations and conduct selective pharmacological studies. This electrophysiological setup evaluates the conduction properties of the sciatic nerve by stimulating the distal end, replicating pain-related signals, and enabling functional analysis of nerve fibers (Figure 1). Key findings include: (1) Conduction velocity: differentiates large fibers (Aβ, Aδ) from small fibers (C), essential for studying nociceptive and mechanosensitive pathways. (2) Amplitude: reflects nerve fiber activity and recruitment potential, which can be altered under conditions such as pain or inflammation. (3) Refractory period: reveals nerve recovery and firing capacity, critical for understanding pain-related repetitive firing. (4) Excitability changes: hyper- and hypo-excitability, which are linked to allodynia and hypoesthesia. By maintaining the viability of the isolated nerve for extended periods, researchers can conduct long-term experiments, enabling detailed investigations into nerve conduction and the impact of therapeutic interventions. This capability is essential for advancing our understanding of neurological diseases and developing new treatments.

## Materials and reagents

**Biological materials**

1. C57BL/J6 or ICR 3–6-month-old mice (Harlan Laboratories)

**Reagents**

1. Potassium chloride (KCl) (Sigma-Aldrich, catalog number: 1.04936)

2. Magnesium sulfate (MgSO_4_) (Bio Lab, catalog number: 13190391)

3. Sodium monophosphate (NaH_2_PO_4_) (Bio Lab, catalog number: 1004921-5)

4. Sodium chloride (NaCl) (Bio Lab, catalog number: 1903059)

5. Sodium bicarbonate (NaHCO_3_) (Frutarom, catalog number: 5553350)

6. Glucose (C_6_H_12_O_6_) (Sigma-Aldrich, catalog number: 1.0837)

7. Calcium chloride (CaCl_2_) (Sigma-Aldrich, catalog number: 1.02382)

8. Pentobarbital (CTS Chemical Industries LTD, catalog number: 2241702)

**Solutions**

1. Stock solutions: 300 mM KCl stock solution, 200 mM MgSO_4_ stock solution, 125 mM NaH_2_PO_4_ stock solution, 200 M CaCl_2_ stock solution, 1,260 mM NaCl stock solution, and 260 mM NaHCO_3_ stock solution (see Recipes)

2. Artificial cerebrospinal fluid (aCSF) (see Recipes)

**Recipes**

**1. Stock solutions**

**Table:** 

Reagent	Final concentration	Quantity	Volume
KCl	300 mM	1.12 g	50 mL
MgSO_4_·7H_2_O	200 mM	2.46 g	50 mL
NaH_2_PO_4_·H_2_O	125 mM	0.86 g	50 mL
CaCl_2_·2H_2_O	200 mM	1.47 g	50 mL
NaCl	1,260 mM	36.82 g	500 mL
NaHCO_3_	260 mM	10.92 g	500 mL

Stocks should be prepared in double-distilled water (DDW) and then filtered. Filtered stocks should be stored at 4 °C for three months and checked periodically for contamination.

**2. Artificial cerebrospinal fluid (500 mL)**

**Table:** 

Reagent	Final concentration	Quantity or Volume
300 mM KCl stock solution	3 mM	5 mL
200 mM MgSO_4_ stock solution	2 mM	5 mL
125 mM NaH_2_PO_4_ stock solution	1.25 mM	5 mL
1,260 mM NaCl stock solution	126 mM	50 mL
260 mM NaHCO_3_ stock solution	26 mM	50 mL
Glucose	10 mM	0.9008 g
200 mM CaCl_2_ stock solution	2 mM	5 mL
DDW	n/a	see note*
Total	n/a	500 mL

On the day of the preparation:

Pour 350 mL of DDW into a beaker containing a stirring bar. Add the KCl, NaH_2_PO_4_, MgSO_4_, NaCl, and NaHCO_3_ from the prepared stocks. Measure the glucose and add it to the solution.

*Note: Do not add CaCl_2_ at this point as it could precipitate. Continue stirring.*

When all powders have dissolved, cover the beaker with parafilm. Oxygenate the solution. Place a clean tube connected to the 95% O_2_, 5% CO_2_ gas tank into the bottle. After 30 min of oxygenation, add CaCl_2_ from the stock, adjust the pH to 7.4 using NaOH, and add DDW to complete the volume to 500 mL. Stir and keep oxygenated.

**Laboratory supplies**

1. Spray bottle with 70% ethanol (Vitamed, catalog number: 7290008082990)

2. 100% ethanol (Sigma-Aldrich, catalog number: 459844)

3. Petri dish 60 mm × 15 mm (Sigma-Aldrich, catalog number: P5481)

4. Sylgard 184 (Dow Corning, catalog number: 3097366-0516, 3097358-1004)

5. Heavy-duty silicone grease (Molykote, Dupont, catalog number: Z273554)

6. Three-compartment chamber (created in-house; see Section A)

7. 95% O_2_ and 5% CO_2_ gas mixture

## Equipment

**Dissection and nerve isolation**

1. Surgical scissors (Fine Science Tools, catalog number: 14200-21)

2. Extra fine Bonn scissors (Fine Science Tools, catalog number: 14084-08)

3. Tissue forceps (Fine Science Tools, catalog number: 11021-12)

4. N° 3 scalpel handle (Sigma-Aldrich, catalog number: S2896)

5. Size 11 carbon steel surgical blade (Swann Morton, Sheffield, England, catalog number: 0203)

6. Vannas scissors (Surgitrac, catalog number: SC81)

7. Straight tying forceps (Surgitrac, catalog number: SC19)

8. Surgical marker (Stericlin, catalog number: 210110)

9. Jeweler’s forceps (Surgitrac, catalog number: SC60)

**Electrophysiology**

1. Digital stereomicroscope (Nikon, model: SMZ745T)

2. Light source, 24 V, 150 W (MXBAOHENG, model: XD-301)

3. Amplifier with headstage (NPI, model: EXT-02F – Extracellular Amplifier)

4. Digitizer (Molecular Devices, model: Digidata 1440A)

5. Current stimulator (AMPI, Isoflex)

6. Faraday cage (WPI, model: AT-3648-FCB)

7. Bare silver wire (diameter: 1 mm; length: 30 mm) for recording and stimulation electrodes (WPI, model: AGW4010)

8. 1 mm × 2.5 mm Ag/AgCl pellet with 70 mm embedded Ag wire (NPI, catalog number: ALA-P-BMP-1)

9. Banana plugs and cable (RS, catalog number: 261-6518)

10. BNC cables (RS, catalog number: 122-2144)

11. PC computer (HP, model: EliteDesk 800 G2 TWR)

12. Four 17.5 mm Eclipse magnets with M6 threaded hole (RS, catalog number: 298-0863)

13. Four M6 screws and bolts (RS, catalog number: 277-604)

14. Warming platform for Petri dish (NPI, model: TC-10/20 Temperature Controller)

15. Temperature controller (NPI, model: TC-10/20 Temperature Controller)

## Software and datasets

1. pClamp (version 10.7, July 2016, Molecular Devices)

2. Clampfit (version 11.3, September 2023, Molecular Devices)

*Note: The software requires a license (Molecular Devices).*

## Procedure

**A. Three-chamber preparation**

**Figure 2.**
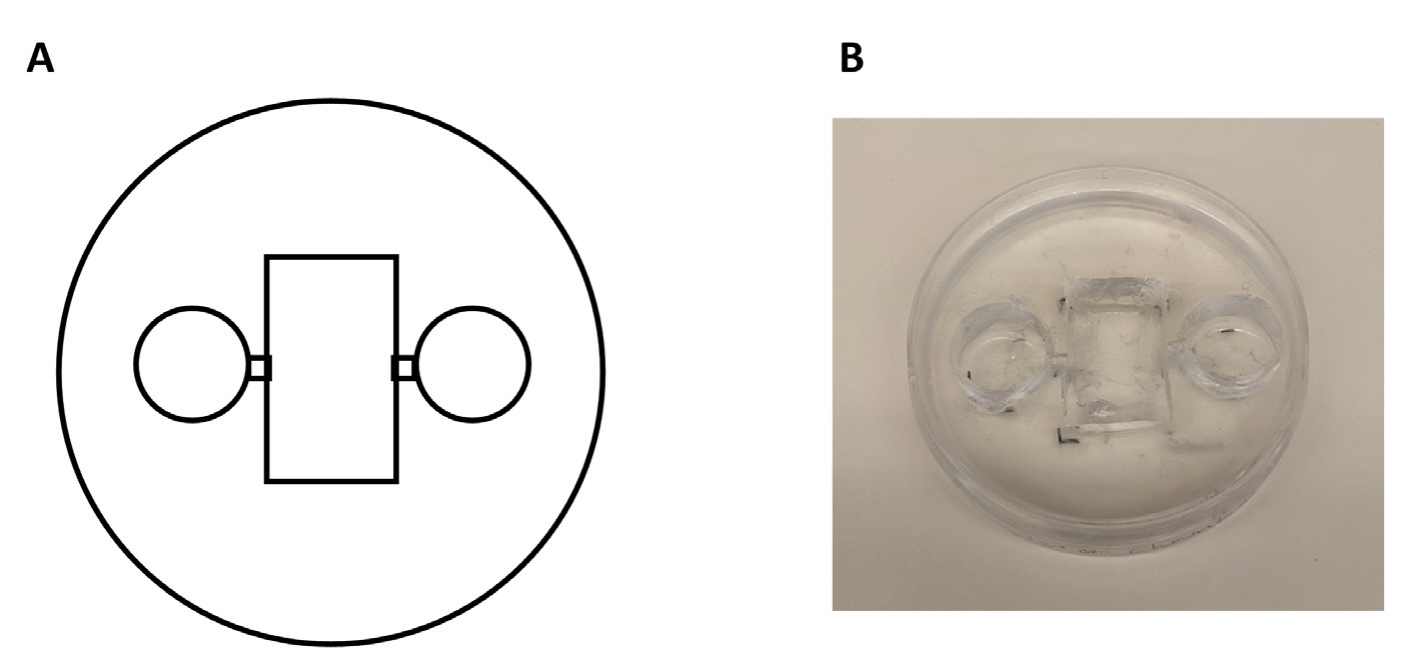
Chamber fabrication process and final product. A. Schematic representation of the chamber construction using a Petri dish and a Sylgard mixture. The Sylgard is poured into the Petri dish and allowed to cure, forming a stable base for the chamber. Additional modifications, such as wells or channels, are drawn on the bottom of the dish and are incorporated during the curing process or cut with a razor blade after polymerization. B. The final chamber product, demonstrating its intended design and functionality.

Day 1:

1. Use a non-permanent marker to delineate the limits of each well on the outside surface of the bottom of a Petri dish. Mark two external wells (10 mm in diameter) and the central chamber (10 × 20 mm) and leave a 2 mm space between the external wells and the central chamber (Figure 2).

2. Prepare the Sylgard mixture:

a. Weigh 30.8 g (30 mL) of Sylgard 184.

b. Add 3.8 g of silicone elastomer (10% of Sylgard 184).

c. Mix the elastomer in a tube by gentle inversion. The total weight should be approximately 33.77 g.

*Note: This quantity is enough to cover one chamber. If making more chambers, prepare using the ratio of 10:1 Sylgard 184 to silicone elastomer.*

3. Set up chambers:

a. Take a 60 × 15 mm plastic Petri dish.

b. Place a test tube (10 mm in diameter) on each lateral marking to mold the lateral wells.

c. Cover the openings of the test tubes with parafilm.

d. Pour a layer of the Sylgard mixture into the Petri dish.

e. Leave the setup at 37 °C overnight.

Day 2:

4. Cut the big chamber:

a. Use a scalpel blade to cut a big rectangle.

b. Remove the cut piece with forceps.

c. Carefully remove the test tubes to create the lateral wells.

5. Create passage/grooves: cut a 1 mm wide passage/groove between the wells using a Gillette razor blade.

6. Clean the plate with 100% ethanol.

7. Add an insulating bottom layer of Sylgard.

a. Prepare the mixture in a 50 mL tube: 5 mL of Sylgard and 500 μL of elastomer (10%).

b. Use a cut Pasteur pipette to add the thin layer of Sylgard onto the central chamber and wells.

**B. Electrode preparation**

For stimulating and recording electrodes, use bare silver wire, a cable, and a banana connector:

1. Remove 2 mm of the isolation layer from both ends of the cable.

2. Spiral 1 cm of silver wire around one of the bare cable ends.

3. Use welding lead to join the cable and wire together.

4. Cover with a 5 mm long shrinkable tube.

5. Complete the isolation by heating the tube with a heat gun.

6. Connect the other cable end to the banana connector.

7. Chloride the recording electrode with HCl or dip it overnight in bleach.

For the Ag/Cl pellet, use a male banana plug, a cable, and the Ag-Cl pellet:

1. As above, remove 2 mm of the isolation layer from both ends of the cable.

2. Spiral the silver wire of the pellet around the cable and weld it.

3. Hermetically seal the connection with shrinkable tube.

**C. Initial preparations for dissection**

**Figure 3.**
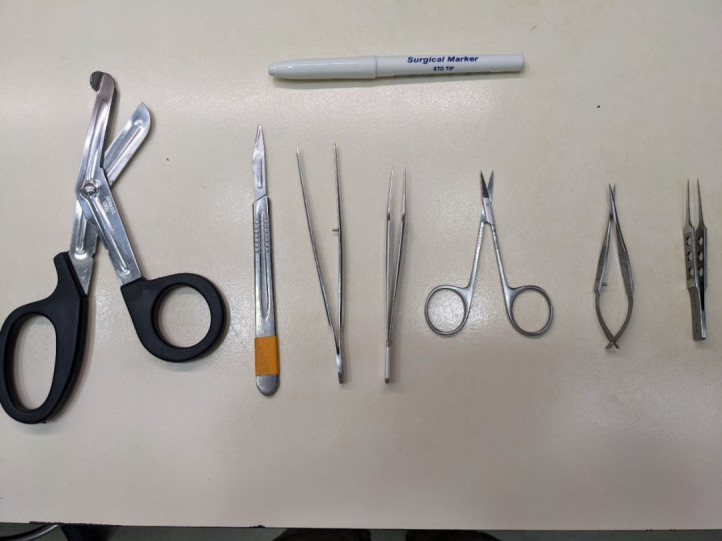
Dissection tools for isolation of the mouse sciatic nerve. Top: surgical marker to mark the distal side of the nerve. From left to right: surgical scissors, scalpel, tissue forceps, jeweler’s forceps, sharp scissors, Vannas scissors, and straight tying forceps.

1. Prior to beginning any procedures, bring the aCSF to room temperature and bubble the solution with a mixture of 95% O_2_ and 5% CO_2_ gas for 15 min.

2. Collect all surgical tools. Disinfect the tools and the working area with 70% ethanol (Figure 3).

3. Before starting the dissection, prepare a 100 × 15 mm Petri dish for the initial collection of the dissected nerves and fill it with aCSF.

*Note: The dissection should be performed in less than 30 min. After dissection, the nerves can be kept in a chamber with continuously bubbled aCSF at room temperature for up to 6 h.*

**Figure 4.**
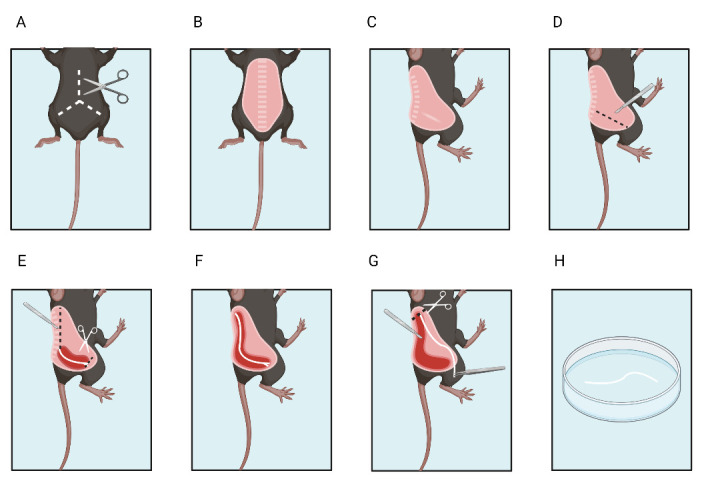
Sciatic nerve dissection. A, B. Remove the fur from the mouse dorsum. C. Place the mouse on the side. D. Make an initial incision around the nerve. E. Cut the distal end. F. Expose the proximal end of the nerve and G. then cut the proximal end. H. Place the excised nerve in artificial cerebrospinal fluid. Created with BioRender.com

4. Terminally anesthetize the animal by intraperitoneal injection of Pentobarbital (2000 mg/kg). Anesthesia can be confirmed by the loss of the hindlimb withdrawal reflex (assessed by pinching the hind paw) and/or the loss of the corneal reflex (assessed by gently touching the eye). Euthanasia is then performed by decapitation.

5. Place the animal in a designated tissue dissection area. Remove dorsal skin with tissue forceps and sharp scissors. Place the mouse on its side (Figure 4A, 4B).

**D. Nerve isolation**

Dissection is performed at room temperature.

1. Expose the sciatic nerve.

a. First, use the scalpel to cut the skin near the sciatic nerve between the sciatic notch and the patella where the nerve is visible through the muscle fascia to expose the nerve (Figure 4D).

b. Begin to isolate the nerve using the tip of the N° 11 scalpel blade by carefully sliding the blade near and along the nerve (Figure 4D).

c. Use a surgical marker to mark the side of the nerve.

d. Continue cutting through the fascia until reaching the distal part of the sciatic nerve where it branches into the tibial and fibular portions.

e. Cut the distal part of the nerve (Figure 4E).

f. Continue to expose the sciatic nerve by following its pathway to its proximal insertion at the spinal cord L3–L4 (Figure 4F).

g. Disjoint the bone junction at the notch by placing the scalpel at a 90° angle with the blade parallel to the spine.

h. Cut through the muscle to fully expose the proximal end of the nerve.

2. Cut and detach the nerve.

a. Once the proximal end is fully exposed (nerve length approximately 20 mm), section it with Vannas scissors (Figure 4G).

b. Hold the distal end of the nerve gently with the fine tying forceps and begin to dissect the nerve out by cutting the remaining connective tissues with Vannas scissors without damaging the nerve or touching the proximal end.

c. Lift the nerve and place it in the Petri dish filled with room temperature aCSF (Figure 4H). Repeat the procedure for the nerve on the other side.

**E. Cleaning the nerve**

*Notes:*

*1. Cleaning is done in a Petri dish at room temperature in aCSF.*

*2. It is crucial to thoroughly remove blood clots, adipose tissues, and connective tissues to maintain consistent resistance across trials.*

1. Under a binocular microscope, carefully clean the nerves to remove all excess branches, muscle, blood clots, and fat.

2. Hold the excess tissue with fine tying forceps and cut it off with Vannas scissors without touching the nerve itself.

3. Once cleaned, place the nerves into continuously oxygenated aCSF at room temperature.

**F. Nerve placement**

1. Pre-fill the bottom of the notches with heavy-duty silicone grease in a U shape.

2. Hold the nerve gently with Jeweler’s forceps or other long forceps. Gently slide the distal side of the nerve through the notch from the proximal end to the distal end.

3. Ensure that there is no overhang on the proximal side to prevent a biphasic signal recording; a slight overhang is acceptable on the distal end.

4. Once the nerve is in place, add silicone grease to the notches and above to keep the nerve firmly in place.

5. Cut any overhang on the proximal side.

6. Add aCSF to the lateral wells and the main chamber and position the chamber on the heating plate.

7. Position the stimulating, recording, and grounding electrodes, as well as the perfusion system.

8. Perfuse the nerve with oxygenated aCSF for 5–10 min while warming to allow the nerve to acclimate slowly.

**G. Initial preparations for nerve stimulation and recordings**

**Figure 5.**
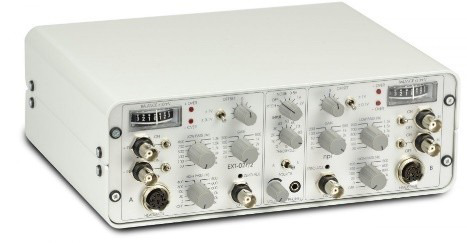
Amplifier. Gain parameters, low pass, and high pass filters can be set manually using the amplifier.

**Figure 6.**
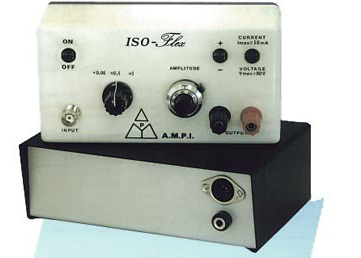
Isoflex stimulator. Stimulation can be given in positive or negative current or voltage. The stimulus max is 10 mA.

1. Setting up the amplifier parameters (Figure 5) and stimulation settings (Figure 6):

a. Amplifier filter settings: To maximize the recording of fast and slow potentials, set the high pass filter to 3 Hz and the low pass filter to 20 kHz.

b. Gain settings: For large fiber assessment, set the gain to 500. For C fiber assessment, increase the gain to 5,000.

**H. Nerve stimulation and recordings**

**Figure 7.**
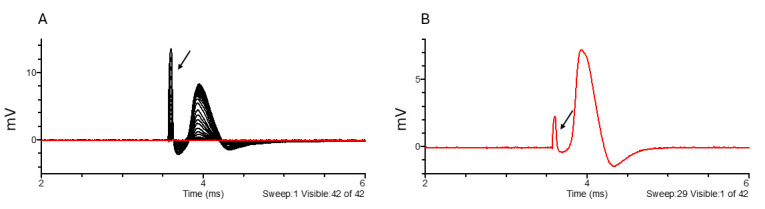
Large fiber recruitment (A) and example of a supramaximal stimulus response to 170 μA stimulation (B). The black arrow points to the stimulus artifact. Black indicates all traces, while red highlights the selected trace.

Data acquisition for large fiber assessment (Figure 7)

1. Recording of compound action potentials (CAP) corresponding to large nerve fibers and assessment of fiber recruitment:

a. Use a 0.1 ms square current pulse of positive or negative polarity (place the stimulator cathode in the central compartment and stimulator anode in the lateral compartment). Set a delay of at least 1 ms before the stimulus to record baseline activity.

b. Start with currents ranging from 0.005 to 1 mA. Interspace each stimulation with a resting period of 200 ms at least.

c. Find the supramaximal stimulus intensity (evoking 85%–90% of the maximal CAP amplitude).

d. Parameters for analysis: peak amplitude, delays between the stimulation and the peak amplitude and half the peak amplitude, duration of the CAP, area under the curve, rise time, threshold of excitation, and recruitment curve.

e. Conduction velocity is calculated from the delays, knowing that the conducting distance is set by the length of the central chamber. For example: 10 mm. CV (m × s^-1^) = 10 (mm)/delay (ms).

**Figure 8.**
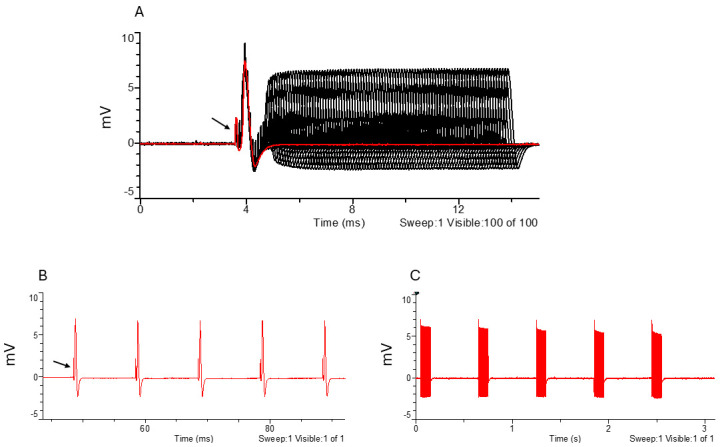
Large fiber refractory period and excitatory parameters. A. Amplitude response to increasing intervals between paired stimuli. B. Response to 100 Hz stimulation frequency. C. Response to high-frequency stimulation frequency (600–1,000 Hz). The black arrow points to the stimulus artifact. Black indicates all traces, while red highlights the selected trace.

2. Assess nerve refractory response (Figure 8A):

a. Use the supramaximal current intensity.

b. Perform paired stimulation and increase the delay between the two paired pulses from 0.1 to 10 ms.

c. Parameters for analysis: maximal amplitude of the second CAP and refractory period curve, which reflects the relative and absolute refractory period of all axonal populations.

3. Assess nerve excitation response and fatiguability (Figure 8B and 8C):

a. Use the supramaximal stimulating current intensity.

b. Stimulate with 100-ms long train pulses of increasing frequency (100–1,000 Hz) interspaced by 0.5 s intervals.

c. Parameters for analysis: amplitude of each CAP within a train relative to the first CAP of the train, amplitude change in response to firing frequency, and slope of amplitude change.

Data acquisition for small unmyelinated fiber assessment

1. Recruit high-threshold C-fibers:

a. Increase gain setting to 5,000.

b. Use a 1-ms long square current pulse. Set a delay of at least 10 ms before the stimulus to record the baseline activity.

c. Use stimulation currents ranging from 0.1 to 1 mA. Interspace each stimulation with a resting period of 1 s at least.

d. Find the supramaximal response.

**Figure 9.**
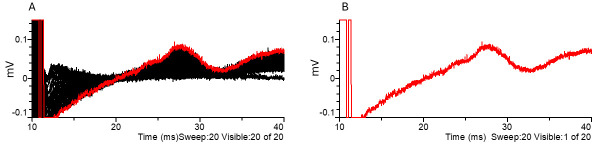
C-fiber parameters. (A) C-fiber amplitude response to increasing stimulus durations and (B) example of C-fiber CAP in response to a supramaximal 1 ms duration stimulus. Black indicates all traces, while red highlights the selected trace. CAP: compound action potential.

*Note: The typical amplitude of an A-fiber-mediated CAP driven by a supramaximal stimulus ranges from 4 to 10 mV. The typical amplitude of a C-fiber-mediated CAP driven by a supramaximal stimulus for a duration of 1 ms ranges from 0.06 to 0.14 mV.*

2. Assess C-fiber response (Figure 9):

a. Use the supramaximal current.

b. Vary the duration of the stimuli from 0.1 to 1 ms.

c. Parameters for analysis: peak amplitude, delays between the stimulation and the peak amplitude (conduction velocity), and stimulus duration/response amplitude curve.

## Data analysis

For data analysis, use Clampfit software. Choose files for comparison (i.e., control and pharmacology file). The criteria for data inclusion are 1) a stable recording over the period of recording, 2) CAP amplitude and conduction velocities within a normal range according to the age, temperature, or condition of the animal, and 3) a stable temperature and flow rate during the period of recording. The criteria for data exclusion are 1) unstable recording, temperature, or flow rate during the recording, 2) abnormally low CAP amplitude or slow conduction velocities according to the age, temperature, or condition of the animal, 3) unusually high or long artifact overlapping with CAP, and 4) biphasic CAP. We recommend assessing at least eight biological replicates (eight nerves) to obtain reliable and significant results.

1. Large fiber recruitment data:

a. Place cursors 3 & 4 before the stimulation artifact (Figure 10) to adjust the baseline to zero in all traces (cursors can be anywhere as long as they are before the CAP and artifact).

b. Place cursor 1 between the stimulus artifact and the CAP (Figure 10).

c. Place cursor 2 after the CAP (Figure 10).

d. Results to analyze include peak amplitude, CAP duration at half width, delay to peak amplitude, delay to half amplitude, and delay to decay half amplitude. Peak amplitude can then be plotted according to stimulation intensity.

**Figure 7. Large fiber compound action potential displayed in Clampfit software.** (A) Example of a file as it appears when opened in Clampfit. (B) Placement of cursors 1 through 4.

For visualization or figure preparation, copy the electrophysiology trace to the graphic design software (PowerPoint, Corel Draw, Adobe illustrator, or CANVAS). Copy the analysis window to the clipboard as a metafile and paste it into your graphic design software; traces can then be de-associated and cropped for final representation. Keep the scale bar and the stimulus artifact. Traces can be overlapped with controls to show drug effects or the effects of neuropathy.

2. Refractory data analysis:

a. Place cursors 3 & 4 before the stimulus artifact of the first CAP to adjust the baseline to zero (cursors can be anywhere as long as they are before the first CAP and artifact).

b. To ease analysis, we suggest subtracting the first CAP from all traces by recording the first CAP alone and removing it using the function *arithmetic*. Quantify the second trace by placing cursor 1 between the artifact and the first CAP, and by placing cursor 2 at the end of the sweeps.

c. If the CAP exceeds the stimulus artifact, analyze the peak amplitude using the same method as for the recruitment data. If the artifact is higher than the CAP, use a filtering method to remove the artifacts or use *event detection* to detect all CAPs.

d. Filtering method: Filter using a low pass to remove high frequencies.

e. Event detection: First, define a template. Then, use *template match threshold* to define the right threshold to detect all events.

f. Results to analyze include peak amplitude that will be plotted according to interpulse intervals.

3. Low train frequency data:

a. Bring cursors 3 & 4 before the stimulus artifact of the first CAP of the train to adjust the baseline to zero (cursors can be anywhere as long as they are before the first CAP and artifact).

b. Place cursors 1 & 2 at the beginning and end of the train of CAPs, respectively.

c. Use *event detection* to create a template and to detect all CAPs between cursors 1 & 2.

d. Assess peak amplitude, slope of peak decay, and time to peak. The number of events will depend on the frequency of stimulations that were applied.

4. High train frequency data:

a. Bring cursors 3 & 4 before the stimulus artifact of the first CAP of the train to adjust the baseline to zero (cursors can be anywhere as long as they are before the first CAP and artifact).

b. Place cursors 1 & 2 at the beginning and end of the train, respectively.

c. Use *event detection* to create a template to detect all CAPs between cursors 1 & 2.

d. Assess peak amplitude, slope of peak decay, time to peak. The number of events will depend on the frequency of stimulations that were applied.

5. C-fiber recruitment and rheobase:

a. Place cursors 3 & 4 before the stimulus artifact to adjust the baseline to zero (cursors can be anywhere as long as they are before the CAP and artifact).

b. Place cursors 1 & 2 around the CAP.

Results to analyze include peak amplitude, CAP duration at half width, delay to peak amplitude, delay to half amplitude, and delay to decay half amplitude. Peak amplitude can then be plotted according to stimulus duration.

## Validation of protocol

This protocol or parts of it has been used and validated in the following research articles:

- Lonigro et al. [15]. Disruption of neurofascin and gliomedin at nodes of Ranvier precedes demyelination in experimental allergic neuritis. Brain (Figure 8, panels A–D).
- Manso et al. [16]. Anti-Neurofascin-155 IgG4 antibodies prevent paranodal complex formation in vivo. J Clin Invest (Figure 5, panels D–G).
- Devaux [17]. The C-terminal domain of ßIV-spectrin is crucial for KCNQ2 aggregation and excitability at nodes of Ranvier. J Physiol (Figure 2, panels A–B).

## General notes and troubleshooting

**General notes**

The peripheral myelinated and unmyelinated fibers are involved in various pathological conditions and are targets for therapeutic strategies aimed at alleviating conduction defects, axonal degeneration, and pain. Therefore, accurately measuring conduction and conduction changes is essential to understand the pathophysiology of peripheral neuropathies and assess the impact of pharmacological interventions. Here, we provide a detailed protocol for measuring nerve conduction and excitability in the adult mouse sciatic nerve. This protocol can be easily adapted to measure conduction in different age groups, and it is suitable for recording from nerves in rats, humans, spinal roots, and other nerve trunks (such as the sural, femoral, and dorsal rami). The size of the recording chamber can be adjusted to fit the nerve and reduced if needed. One notable benefit of this procedure is that it avoids the use of suction electrodes and the need to maintain negative pressure, which can compromise recording stability and signal quality.

1. With proper cleaning to remove blood clots, nerves can remain functional for several hours in oxygenated aCSF at room temperature.

2. Unlike central nervous system nerves, peripheral nerves should not be kept on ice, as this may impair nerve activity.

3. In this protocol, we recommend preserving the nerve epineurium and perineurium to reduce distortion of nerve activity. When studying nerve activity in neuropathy models, we strongly advise keeping the epineurium/perineurium intact. Careful dissection is crucial to prevent deterioration of nerve activity. Removing the perineurium is a complex and time-consuming procedure that may also affect nerve function. However, for pharmacological testing, removing the epineurium/perineurium can enhance drug access and washouts. Alternatively, spinal nerve roots, which lack epineurium and perineurium, can be used for drug testing.

4. We recommend using either a Sylgard-coated homemade chamber or a Plexiglas chamber rather than a 3D-printed chamber, as the latter may have micro holes that interfere with the quality of nerve stimulation and recording. However, a 3D-printed matrix of the internal wells can be used to mold a Sylgard chamber.

5. In this protocol, we suggest using a peristaltic pump to precisely control the flow rate of aCSF, which in turn regulates temperature. Temperature is a critical factor in recording peripheral nerve activity as it significantly affects conduction speed, compound action potential amplitude, excitability, refractory period, and responses to stimulus trains. Therefore, it is essential to control perfusion temperature carefully. For pharmacological studies, a peristaltic pump allows for the recirculation of the flowthrough into the perfusion. However, keep in mind that peristaltic pumps can increase electrical noise or generate action potential–like artifacts, so gravity perfusion is recommended when recording spontaneous nerve activity.

6. Although nerve activity remains stable for several hours, we advise completing all recordings within a few hours to minimize the effects of Wallerian degeneration, which can reduce recording quality and lead to activity rundown.

7. We recommend monitoring nerve activity for at least 20 min before beginning pharmacological testing.

**Troubleshooting**

Problem 1: Baseline shifts.

Possible causes: Electrodes need re-chlorination, noise in setup.

Solutions: Re-chlorinate recording electrodes, identify where the noise is coming from, and ground it if possible. When correctly chlorinated, electrodes should appear gray, and oxidized electrodes will appear black.

Problem 2: Small CAP amplitude.

Possible causes: Possible leakage (shortcut) in the recording chamber or the nerve has been damaged during dissection.

Solutions: Replace the nerve with a new Vaseline grease seal or change the nerve.

Problem 3: Bipolar CAP.

Possible cause: The nerve end is overhanging on the recording side.

Solutions: Adjust the nerve position or clamp the nerve end at the recording side.

Problem 4: Noise.

Possible cause: Improper setting of the electrophysiology machinery.

Solutions: The digitizer, amplifier, and computer should be plugged into separate outlets far from one another. The computer should be plugged in far from the other devices. All apparatus, including the Faraday cage, should be grounded together. Verify that the ground electrode is in the central well and connected to the headstage. Clean the electrodes. Grease may cover one electrode. Verify that grease is not covering the nerve end.

Problem 5: No signal.

Possible causes: The nerve is damaged, or there is an issue with an electronic device.

Solutions: Replace or use another nerve. To test that the digitizer is running correctly, send a test pulse stimulation from the Clampex software. Make sure that the power supply unit is connected to the digitizer and has the correct input/output voltages/current. Test the stimulator electrodes by increasing the stimulation current duration and making sure the stimulation artifact matches the current given. Test the headstage with the test generator (NPI). A given square pulse should result in a square pulse. If the signal from the test generator is clean and there is still noise, then most likely it is coming from somewhere other than the amplifier. Clean the electrodes. Grease may cover the electrodes and impair recording or stimulation.

Problem 6: Unstable signal.

Possible causes: The temperature is improperly controlled, the perfusion flow is unstable, or the nerve has been damaged.

Solutions: Carefully monitor the perfusion temperature with a temperature controller. If the temperature rises above 37 °C, it may irreversibly damage nerve activity. Verify that the perfusion flow is stable. Monitor nerve activity for at least 20 min. If the activity is rundown while the temperature and perfusion are stable, then the nerve must have been damaged and should be replaced.
